# Tenascin-C deficiency impairs alveolarization and microvascular maturation during postnatal lung development

**DOI:** 10.1152/japplphysiol.00258.2019

**Published:** 2020-02-20

**Authors:** Sonja I. Mund, Johannes C. Schittny

**Affiliations:** Institute of Anatomy, University of Bern, Bern, Switzerland

**Keywords:** cell proliferation, lung developmental, microvascular maturation, pulmonary alveolarization, tenascin-C deficiency

## Abstract

After the airways have been formed by branching morphogenesis the gas exchange area of the developing lung is enlarged by the formation of new alveolar septa (alveolarization). The septa themselves mature by a reduction of their double-layered capillary networks to single-layered ones (microvascular maturation). Alveolarization in mice is subdivided into a first phase (*postnatal days 4–21*, classical alveolarization), where new septa are lifted off from immature preexisting septa, and a second phase (*day 14* to adulthood, continued alveolarization), where new septa are formed from mature septa. Tenascin-C (TNC) is a multidomain extracellular matrix protein contributing to organogenesis and tumorigenesis. It is highly expressed during classical alveolarization, but afterward its expression is markedly reduced. To study the effect of TNC deficiency on postnatal lung development, the formation and maturation of the alveolar septa were followed stereologically. Furthermore, the number of proliferating (Ki-67-positive) and TUNEL-positive cells was estimated. In TNC-deficient mice for both phases of alveolarization a delay and catch-up were observed. Cell proliferation was increased at *days 4* and *6*; at *day 7*, thick septa with an accumulation of capillaries and cells were observed; and the number of TUNEL-positive cells (dying cells or DNA repair) was increased at *day 10*. Whereas at *days 15* and *21* premature microvascular maturation was detected, the microvasculature was less mature at *day 60* compared with wild type. No differences were observed in adulthood. We conclude that TNC contributes to the formation of new septa, to microvascular maturation, and to cell proliferation and migration during postnatal lung development.

**NEW & NOTEWORTHY** Previously, we showed that the extracellular matrix protein tenascin-C takes part in prenatal lung development by controlling branching morphogenesis. Now we report that tenascin-C is also important during postnatal lung development, because tenascin-C deficiency delays the formation and maturation of the alveolar septa during not only classical but also continued alveolarization. Adult lungs are indistinguishable from wild type because of a catch-up formation of new septa.

## INTRODUCTION

Starting from the lung buds (lung anlage) the conducting airways and parts of the respiratory airways are formed by continuous cycles of branching and growth into the surrounding mesenchyme [branching morphogenesis (65, 83)]. During the stage of alveolarization the gas exchange surface area is enlarged by the lifting off of new alveolar septa from the preexisting septa. The newly formed septa increase in height and subdivide the existing air spaces into smaller units (septation), called alveoli. During the lifting off of new alveolar septa, one leaflet of the existing double-layered capillary network within the existing septa folds up and gives rise to a new double-layered capillary network inside the newly formed septa (3, 6, 7, 65, 87). Depending on the point of view, this process is called either alveolarization or septation. Whereas alveolarization focuses on the formation of new air spaces (alveoli), septation focuses on the formation of new walls (septa) that subdivide the existing air spaces. To optimize gas exchange, the double-layered capillary networks of all septa are reduced to a central, single-layered one by capillary fusion, and the former central layer of connective tissue is reduced to a thin fibrous meshwork interwoven with the capillaries (microvascular maturation). Alveolarization and microvascular maturation start in parallel around *postnatal* *day 4* in mice and rats and continue until young adulthood (50, 59, 65, 70). The stage of alveolarization can be subdivided into two phases. Classical alveolarization (*days 4–21* in mice and rats) is characterized by the lifting off/folding up of new septa from immature preexisting septa containing a double-layered capillary network, whereas during continued alveolarization [*day 14* to adulthood (roughly *days 36–60*) in mice and rats], new septa are lifted off/folded up from mature preexisting septa containing a single-layered capillary network (1, 50, 70). In humans, alveolarization is considered to start before birth and last up to young adulthood, and microvascular maturation is regarded to last until 2–3 yr of age (65, 66, 83).

Although the structural mechanism of alveolarization/septation has been well established, knowledge about the cellular processes and molecular signals guiding the lifting off of new septa is still limited (61, 67). It is well recognized that smooth muscle cells, elastic fibers, and collagen fibrils appear concentrated at the free edges of the existing and newly forming septa throughout septation. The presence of these three components seems to be crucial for the formation of new alveolar septa (20, 61, 67). In addition, the importance of cell proliferation for the lifting off of new septa was emphasized by the finding that in rats the rate of cell proliferation of all major cell types increased exactly in parallel with the beginning of classical alveolarization (39, 45). The absolute number of fibroblasts and epithelial cells is later diminished by apoptosis. In rats a peak of programmed cell death was detected at the end of the third postnatal week (45, 68). To the best of our knowledge, programmed cell death has so far never been followed during postnatal lung development in mice but is expected to be similar.

It is well acknowledged that the extracellular matrix plays an important role in regulating the behavior of cells that contact it. During prenatal lung development there is much evidence that different components of the extracellular matrix, such as tenascin-C, elastin, fibronectin, and different laminin isoforms, have unique functions in the regulation of branching morphogenesis (61). However, less is known about the role of the extracellular matrix in alveolarization and microvascular maturation. Tenascin-C (TNC) is a large, hexameric glycoprotein of the extracellular matrix. It is transiently expressed during organogenesis, where it is especially prominent at mesenchymal-epithelial interaction sites and along pathways of migrating cells. TNC is markedly reduced in adult tissues but reappears under pathological conditions such as inflammation and tumorigenesis (8, 9, 21, 23, 61, 78, 84). During prenatal lung development, TNC accumulates in the basement membranes and mesenchyme surrounding the branching and growing tips of the bronchial tree (41, 85, 88). At this location it contributes to the control of branching morphogenesis (60). At the beginning of classical alveolarization, TNC appears to be concentrated at the tips of the newly forming septa in parallel with smooth muscle cells, elastic fibers, and collagen fibrils. During the following postnatal lung development and during adolescence, TNC expression declines to rarely detectable levels (38, 53, 62, 85). TNC expression is upregulated by different growth factors and cytokines, as well as by mechanical stress (8, 15). It was shown to be downregulated by glucocorticoids (36, 58) and also in surgically induced congenital diaphragmatic hernia (81).

Antibody perturbation assays and tissue culture studies have suggested multiple functions for TNC (12). TNC has been shown to inhibit adhesion to fibronectin of most cells in culture, but for some cells, it functions as adhesion substrate. Therefore, it has been classified as an adhesion-modulating protein (10, 54, 63). Furthermore, depending on the cell type, TNC has been demonstrated to promote or inhibit cell migration and cell proliferation and to modulate cell shape (18, 36, 46). Given the multiplicity of functions that have been suggested for TNC by in vitro studies, it was rather surprising when TNC null mice were initially reported to show no abnormalities. By the same token, no mechanism of compensation for the loss of TNC was found (5, 18, 24, 47, 62, 80). Looking in more detail, it became evident that TNC knockout mice show subtle phenotypes, e.g., *1*) behavioral abnormalities (26, 40), *2*) a reduced hematopoietic activity of bone marrow cells (52), *3*) an impaired healing of corneal wounds in corneas that were exposed to mechanical stress (48), *4*) a suppression of the formation of fibrous adhesions after injury of the temporomandibular joint (73), and *5*) a reduced Wnt/β-catenin signaling combined with a reduced proliferation and migration of stem cells in whisker follicle stem cell niches (32, 79).

Developmental alterations have also been reported (13). For example, *1*) fetal lung organ cultures of TNC null mice showed a reduction in the number of branches, whereas the growth of the lung explants was not altered (60), and *2*) increased migration and reduced proliferation of neural precursor cells were detected during the development of the central nervous system (40).

Although it is well recognized that in the developing lung the expression of TNC peaks at the start of the first phase of alveolarization (classical alveolarization), the effect of TNC deficiency during this phase has never been investigated so far. The aim of the present study was to provide this information. Therefore, we followed alveolarization/septation and microvascular maturation in the TNC null mouse strain generated by Forsberg et al. (24) and in matched wild-type mice using morphological and stereological methods. In addition, the extent of cell proliferation and of terminal deoxynucleotidyl transferase (TdT)-mediated dUTP nick end labeling (TUNEL)-positive cells was compared between TNC null and wild-type lungs.

## MATERIALS AND METHODS

### Animals and Tissues

Lungs from the TNC null mouse strain “Tnc tm1Ref” of Forsberg et al. (24) and from 129/SV wild-type control animals were obtained between *postnatal days 2* and *86* as described in the following. For every data point, *n* = 3–8 male animals were used (see figure legends), because the experiments were done at a time when the ethics committee asked for one sex only to reduce the number of animals necessary for the study. The animals were housed in the central animal facility of the University of Bern under a 12:12-h day-night cycle. They received water and food ad libitum. The animals were deeply anesthetized using a mixture of medetomidine, midazolam, and fentanyl (22) and afterward euthanized by exsanguination during the removal of the lung. After abdomen and thorax of the deeply anesthetized mice were opened, the air space was filled via tracheal instillation with freshly prepared 4% paraformaldehyde in phosphate-buffered saline (PBS; 10 mM sodium phosphate, containing 127 mM sodium chloride, pH 7.4) at a constant pressure of 20 cmH_2_O. At this pressure, the lung reaches roughly its total lung capacity. To prevent a recoiling of the lung, the pressure was maintained at least for 2 h at 4°C. For the immunohistochemical investigation, the pulmonary blood vessels were perfused beforehand with PBS (10 mM sodium phosphate, containing 127 mM sodium chloride, pH 7.4), containing 5 U/mL heparin, 10 mg/mL procaine, and 10 mM EDTA (Fluka Chemie AG, Buchs, Switzerland).

Handling of the animals before and during the experiments, as well as the experiments themselves, was approved and supervised by the Swiss Agency for Environment, Forests and Landscape and the Veterinary Service of the Canton of Berne. For ethical reasons we were obliged to keep the number of animals as low as possible. Therefore, we used the left lung for the stereological studies, the right lower lobe for imaging, and the remaining lobes for histochemical staining. According to Zeltner et al. (86) and Barré et al. (4) the lobes represent a representative sample of the entire lung.

For light microscopical morphometry as well as for TUNEL assay and Ki-67 staining the left lung was dehydrated en bloc in a graded series of ethanol and embedded in paraffin using Histo-Clear (Life Science International, Frankfurt, Germany) as intermedium. A series of step sections of 4.5-µm thickness were obtained perpendicular to the longitudinal axis of the left lung at 10–13 equally spaced locations. The gap between the locations (length of the step) was constant for all lobes obtained at the same postnatal day but increased with the size of the lobes. The first location was determined as follows. The blocks were cut until first pieces of lung appeared in the sections. Afterward a randomly selected number of sections was discarded before the first step section was taken/the first location was reached. This number was smaller than the number of sections between two equally spaced locations. The sections were transferred onto silanized microscope slides and air-dried overnight at 37°C. Sections used for light microscopical morphometry were stained with fuchsine.

Approximately 40 images were taken from all serial sections of the left lung of each animal according to a systematic random sampling scheme (19). Images were recorded using a Leica DM RB light microscope (Glattbrugg, Switzerland) equipped with a motorized Märzhäuser XY stage (Wetzlar, Germany) and a JVC 930 three-chip color video camera (Oberwil, Switzerland) and the software analySIS (Olympus Soft Imaging Solutions, Münster, Germany). The estimation of the volume density of the lung parenchyma, the septal surface area density, the length of the free septal edge, and the number of TUNEL-positive cells was done at a final magnification of ×250, whereas for the estimation of the number of proliferating cells and the total number of cells a final magnification of ×870 was used.

For transmission electron microscopy and synchrotron radiation X-ray tomographic microscopy the right upper and right lower lobes were diced into tissue cubes of ~2-mm edge length. The tissue blocks were postfixed with 2.5% glutaraldehyde in 0.03 M potassium phosphate buffer (pH 7.4, osmolarity 360 mosM) for at least 48 h at 4°C, stained for 1 h in 1% Na-cacodylate-buffered osmium tetroxide solution (osmolarity 350 mosM, pH 7.4), and stained for another 2 h in 0.5% uranyl acetate solution. After dehydration in a graded series of ethanol the tissue blocks were embedded in Epon 812 (68).

For transmission electron microscopy, five Epon-embedded tissue blocks of the right upper lobes were randomly taken, and ultrathin sections (80–90 nm) were cut using a Reichert–Jung Ultracut microtome. Sections were double stained with lead citrate (56) and uranyl acetate (25). One section per block was viewed in a Philips 400 transmission electron microscope. Approximately 25 images per section were taken according to a systematic random sampling scheme (19) by a Morada camera (Soft Imaging System; Olympus Soft Imaging Solutions, Münster, Germany) and the software iTEM (Olympus Soft Imaging Solutions). Stereological measurements were done at a final magnification of ×3,400.

For synchrotron radiation X-ray tomographic microscopy, five blocks of the right lower lobes were randomly taken, shaped down to rods of a diameter of 1.3 mm on a watchmaker’s lathe, and glued onto a rodlike holder with a diameter of 3.0 mm. Special care was taken that they were mounted perpendicularly to the surface of the holder to fit exactly into the window of the camera.

### Immunohistochemistry

Immunohistochemistry was applied to stain proliferating cells with anti-Ki-67, a marker of cell proliferation (71), and to stain TUNEL-positive cells by performing the TdT-mediated dUTP nick end labeling assay adapted from Gavrieli et al. (29).

#### Anti-Ki-67 staining.

As described by Schittny and colleagues (45, 68), paraffin sections were cooked in a household pressure cooker in Target Retrieval Solution (DAKO, Glostrup, Denmark) for 13 min at 2 bar, blocked with Tris-buffered saline containing 100 mg/mL casein (Sigma), and incubated overnight at 4°C with the monoclonal rat anti-mouse Ki-67 antibody (Clone Tec-3, DAKO; diluted 1:50 in antibody diluent, DAKO). Immunoreactivity was detected using the biotinylated polyclonal rabbit anti-rat antibody (DAKO; diluted 1:200 in antibody diluent, DAKO), streptavidin-biotin horseradish peroxidase complex (DAKO), and 3-amino-9-ethylcarbazole (Sigma) as a substrate. The nuclei were counterstained with Mayer’s hematoxylin (VWR, Darmstadt, Germany).

#### TUNEL assay.

As described by Schittny and colleagues (45, 68), paraffin sections were digested with 3.6 μg/mL proteinase K (21°C, 10 min) and incubated with terminal transferase reaction solution, containing 9 mM digoxigenin-11-dUTP and 0.165 U/mL enzyme (Roche, Rotkreuz, Switzerland), for 40 min at 37°C. The incorporated digoxigenin was detected using an alkaline phosphatase-labeled anti-digoxigenin antibody (Roche; diluted 1:1,000 in blocking reagent for nucleic acid hybridization and detection, Roche) and 4-nitro-blue-tetrazolium-chloride (Roche Diagnostics, Mannheim, Germany).

Negative controls were performed with nonspecific mouse IgG (Ki-67 staining) or by omitting the terminal transferase reaction solution (TUNEL). No or only little nonspecific background was observed in all negative controls. In addition, Ki-67 was observed as nuclear staining only.

### Light Microscopical Morphometry

After fixation the volumes of the left lungs were first measured by water displacement (64). After embedding in paraffin and sectioning, the lung volumes were estimated by the Cavalieri method (33, 49). Both volumes were used to calculate the shrinkage for every lung to correct for the shrinkage. The volume density of the lung parenchyma (air spaces and septal tissue, excluding bronchi, bronchioles, and blood vessels >20 µm in diameter) was estimated by point counting.

The surface density of the alveolar septa was estimated by intersection counting. The absolute values were calculated as the product of the surface density and the lung volume for each animal and each time point (33, 82).

The length density and length of the free septal edge were estimated stereologically as described and applied by Schittny and colleagues (50, 58, 70, 77). Briefly, this approach is based on the following two principles. First, any length appearing in three-dimensional (3-D) space may be stereologically estimated by counting the number of points cutting the plane of 2-D sections (33, 82). Second, in 3-D space every air space possesses one entrance ring, which is represented by the free edges of the alveolar septa. Because the free septal edges are recognized as tips of the cut septa in 2-D sections, their length density was estimated by counting the number of the tips of the cut septa in a reference area on paraffin sections. By simple enlargement of the lung, without the addition of new septa, the length density of the free edges of the alveolar septa will decrease, because a volume increases by a factor of *x*^3^, whereas a length increases only by a factor of *x*^1^. This kind of growth follows the principle of isometric scaling and geometric similarity, meaning that proportional relationships are preserved. For example, when the volume increases by a factor of 8, the surface increases by a factor of 4 and the length by a factor of 2. This principle, the square-cube law (27), was most likely first described by Galileo Galilei in 1638. To calculate the length of the free septal edge that was newly formed in addition to the isometric scaled growth of the lung, we mathematically corrected the growth-induced decrease of the length density by multiplying the length density by a factor of ^3^√ (V*_x_*/V_0_)^2^ (thereby V*_x_* represents the parenchymal lung volume at the time point *X*, and V_0_ represents the volume at the start of the growth). The resulting “growth-corrected length density” stays constant throughout isometric scaled growth of the lung parenchyma but shows an increase if new septa are formed. Therefore, the increase in the growth-corrected length density was taken as a measure for the anlage of new alveolar septa as follows. The growth-corrected length density at a given day was divided by the growth-corrected length density at *day 4* and multiplied by 100 to express the result as a percentage. Therefore, the anlage of new septa is given as the increase in the septa present at *day 4*.

The number of proliferating or TUNEL-positive cells as well as the total number of cells was estimated using the physical disector principle (33, 76). The disector was kept constant at 9 μm.

### Electron Microscopical Morphometry

The fraction of the alveolar surface area characterized by a single- or double-layered capillary network or an atypical appearance with >2 capillary layers was estimated by intersection counting (33, 82). In addition, the thickness of the septum was measured perpendicular to the surface of the septum at each intersection. Intersections with lung epithelium adjacent to nonparenchymal structures were not taken into account (82).

### Synchrotron Radiation X-Ray Tomographic Microscopy and Visualization

Five samples of each time point were scanned at the Tomographic Microscopy and Coherent Radiology Experiments (TOMCAT; X02DA) beamline at the Swiss Light Source of the Paul Scherrer Institute, Villigen, Switzerland (75). The energy was tuned to 12.398 keV (corresponding to an X-ray wavelength of 1 Å). After penetration of the sample, X-rays were converted into visible light by a thin Ce-doped yttrium aluminum garnet (YAG) scintillator screen (Crismatec Saint-Gobain, Nemours, France). Projection images were further magnified by diffraction-limited microscope optics and finally digitized by a high-resolution charge-coupled device camera (Photonic Science, East Sussex, United Kingdom; 74). The optical magnification was set to ×10 and on-chip binning was selected to improve the signal-to-noise ratio, resulting in isotropic voxels of 1.4^3^ μm^3^ for the reconstructed images. For each measurement, 1,500 projections were acquired along with dark and periodic flat field images at an integration time of 100 ms each (30, 31, 42–44). Data were postprocessed and rearranged into flat field-corrected sinograms online. Reconstruction of the volume of interest was performed on a 24-node Linux personal computer farm using highly optimized filtered back projection routines. We used a global thresholding approach for surface rendering. For 3-D visualization and surface rendering we used the software Imaris (Bitplane AG, Zürich, Switzerland) on an Athlon 64 3500-based personal computer. To enhance the contrast between air and lung tissue and to smooth the images, we applied the gamma correction tool using the software Adobe Photoshop C53 version 10.0 (Adobe Systems, Microsoft Windows Media Technologies).

### Statistical Analysis

The Kolmogorov–Smirnov test was applied to assess the Gaussian distribution of the data. Differences between groups were assessed by one-way analysis of variance followed by Bonferroni–Holm-corrected post hoc *t* tests (2, 57). Statistical significance was defined as α < 0.05. For all morphometrical measurements, three to eight male animals per time point were used (see figure legends).

## RESULTS

### Morphological Observation in 3-D Visualizations of the Lung Parenchyma

**Fig. 1.**
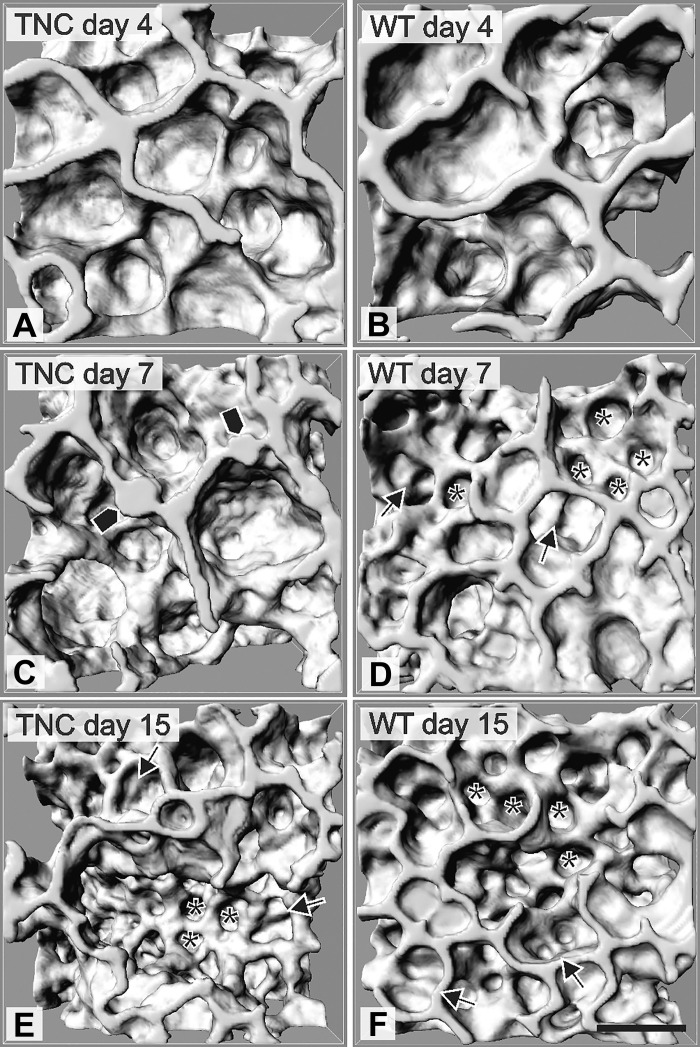
Three-dimensional visualizations of the terminal air spaces. On *postnatal day 4* the lung parenchyma of wild-type (WT) and tenascin-C (TNC) null mice consisted of large terminal air spaces (*A* and *B*). In wild-type mice the start of alveolarization is characterized by the formation of new septa (arrows) and alveoli (*) on *day 7* (*D*). In TNC null lungs, focal areas of atypical thickened septa were detected (bullets in *C*) on *day 7*, which are indicative for a halted alveolarization. At *postnatal* *day 15* the differences disappeared (*E* and *F*). Scale bars, 50 μm; visualizations are based on synchrotron-based X-ray tomographic microscopy.

To study the effect of TNC deficiency during postnatal lung development, we morphologically compared the 3-D structure of the terminal air spaces between TNC null and wild-type lungs during the phase of classical alveolarization. As method, we used 3-D visualizations that were obtained by X-ray tomographic microscopy. At *day 4* the lungs of TNC-deficient and wild-type animals showed a similar appearance. The lung parenchyma was characterized by large terminal air spaces (saccules) in both groups (Fig. 1, *A* and *B*). At *day 7*, newly formed septa and alveoli were detected in wild-type lungs indicating that alveolarization is ongoing (Fig. 1*D*). TNC null lungs of the same age appeared to be in a state analogous to *day 4* but showed focal areas of atypically thickened septa (Fig. 1*C*, bullets). At *postnatal* *day 15* we were not able to observe any structural differences between the two groups by morphological inspection of the 3-D visualizations at light microscopical resolution (Fig. 1, *E* and *F*).

### Stereological Estimations

**Fig. 2.**
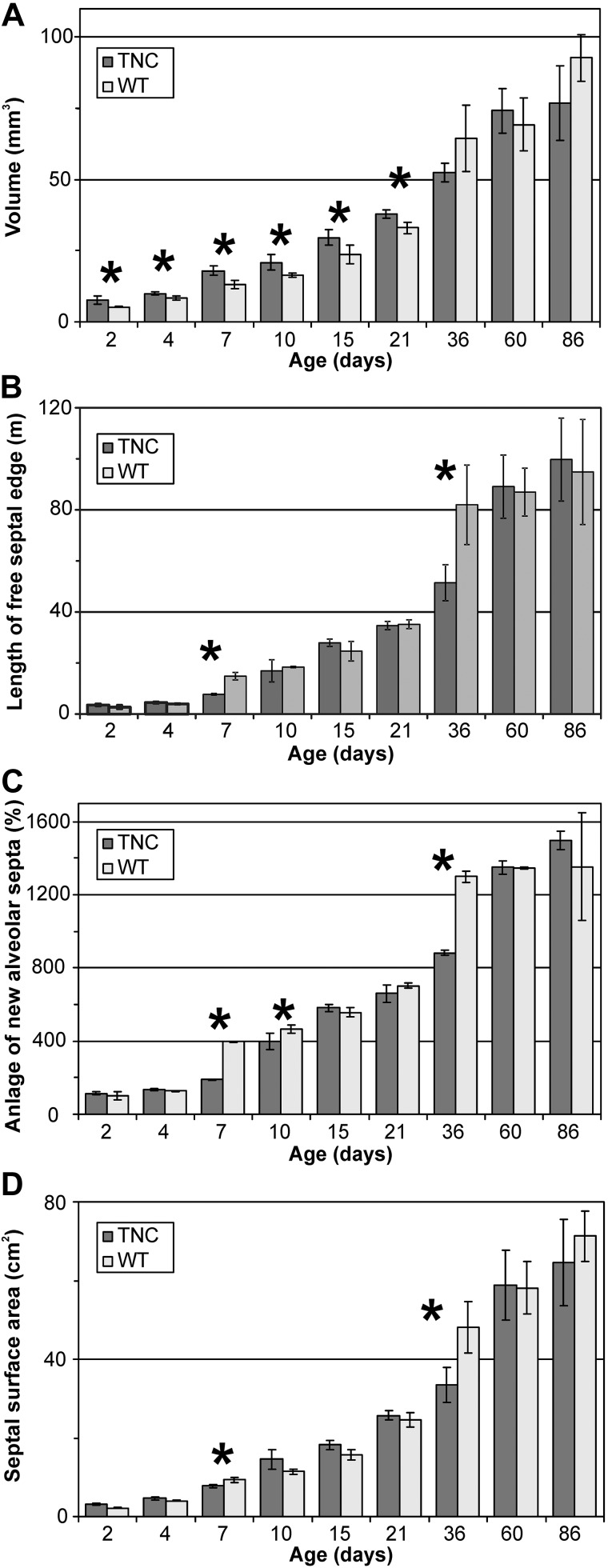
Lung volume, total length of the free septal edge, anlage of newly forming alveolar septa, and total surface area. The lung volumes (*A*), the total length of the free septal edge (*B*), the anlage of newly forming alveolar septa (*C*), and the septal (alveolar) surface area (*D*) were stereologically estimated. The anlage of newly formed septa is normalized to *day 2* and equal to the newly formed length of the free septal edge. The lung volumes of tenascin-C (TNC) null animals were increased by ~20% between *days 2* and *21* (*A*). In TNC null lungs, classical alveolarization started delayed after *day 7* (*B*–*D*). Furthermore, the formation of new alveolar septa was markedly reduced between *days 21* and *36*, but alveolarization was prolonged to *day 60* (*B* and *C*). The septal surface area of TNC-deficient animals was decreased at *days 7* and *36* (*D*). Error bars indicate SD; *n* = 5–8 lungs of male mice per time point and genotype. WT, wild type. *Statistically significant, α < 0.05.

To verify our observations, the lung volumes, the anlage of new alveolar septa, and the septal surface area were quantified and compared between wild-type and TNC null animals. The lung volumes of TNC-deficient animals were increased by 10–20% between *days 2* and *21* (Fig. 2*A*). We did not observe any differences regarding the body weight of TNC null versus wild-type animals. Thus, the specific lung volume (lung volumes per body weight) of TNC null animals was larger than that of wild-type mice between *days 4* and *21* (data not shown). By following the anlage of new alveolar septa and alveolar surface area, we observed that the first and second phases of alveolarization (classical and continued alveolarization) were delayed. Alveolarization started delayed after *day 7* in TNC-deficient lungs, was compensated at *days 15−21*, was again delayed at *day 36*, and was again compensated at *day 60* and afterward (Fig. 2, *B* and *C*).

**Fig. 3.**
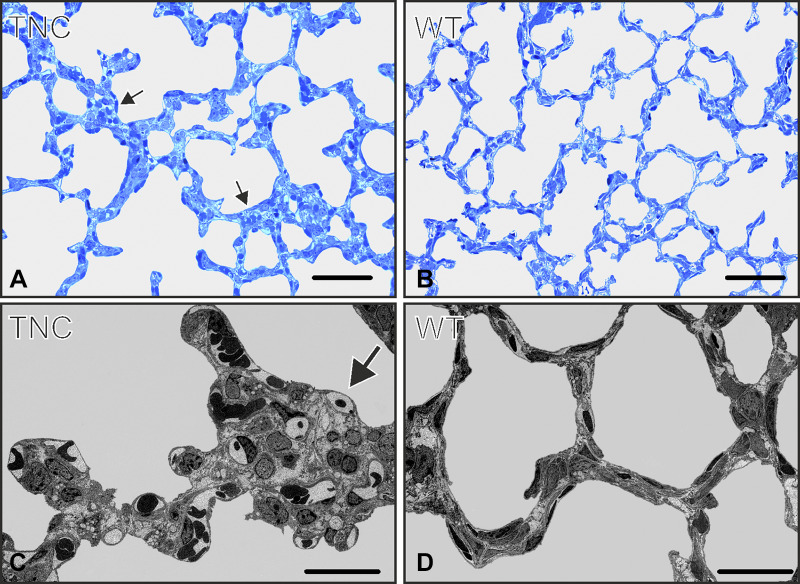
Morphological observations on light and electron microscopical images at *postnatal day 7*. The morphological appearance of the inter-air space septa of wild-type (WT) and tenascin-C (TNC) null lungs was compared on light (*A* and *B*) and electron microscopical images (*C* and *D*). We observed focal areas with atypical thickened septa in TNC null lungs at *postnatal day 7* (arrows). The focally thickened areas showed an abnormal structure with an accumulation of capillaries and connective tissue, as well as an increased cellularity, but no epithelial cells inside the thickening. Scale bars, 50 μm in *A* and *B*; 20 μm in *C* and *D*.

**Fig. 4.**
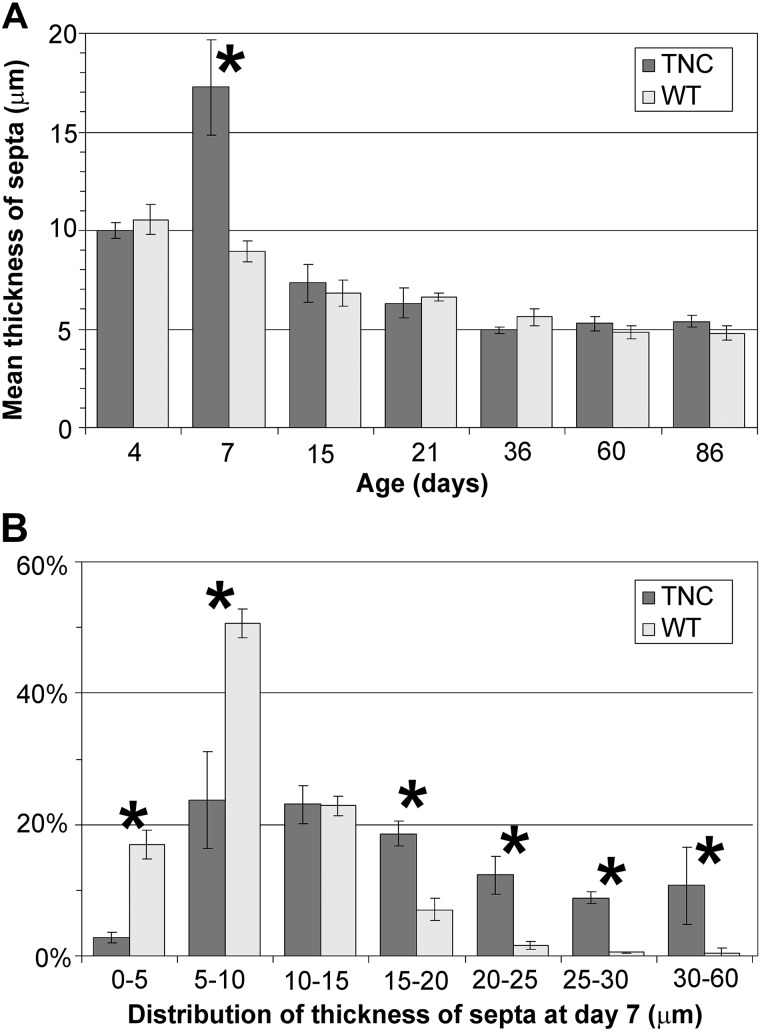
Thickness of septa. The thickness of the septa was measured on electron microscopical lung images as shown in Fig. 3, *C* and *D*. At *day 7* the mean septal wall thickness of the tenascin-C (TNC) null mice was increased by 100% compared with wild-type (WT) mice (*A*). *B* shows a histogram of the thickness measured at *postnatal day 7* using a class width of 5 μm. In TNC-deficient lungs a broader distribution and a shift to thicker septa were observed. Error bars indicate the SD; *n* = 5 lungs of male mice per time point and genotype. *Statistically significant, α < 0.05.

**Fig. 5.**
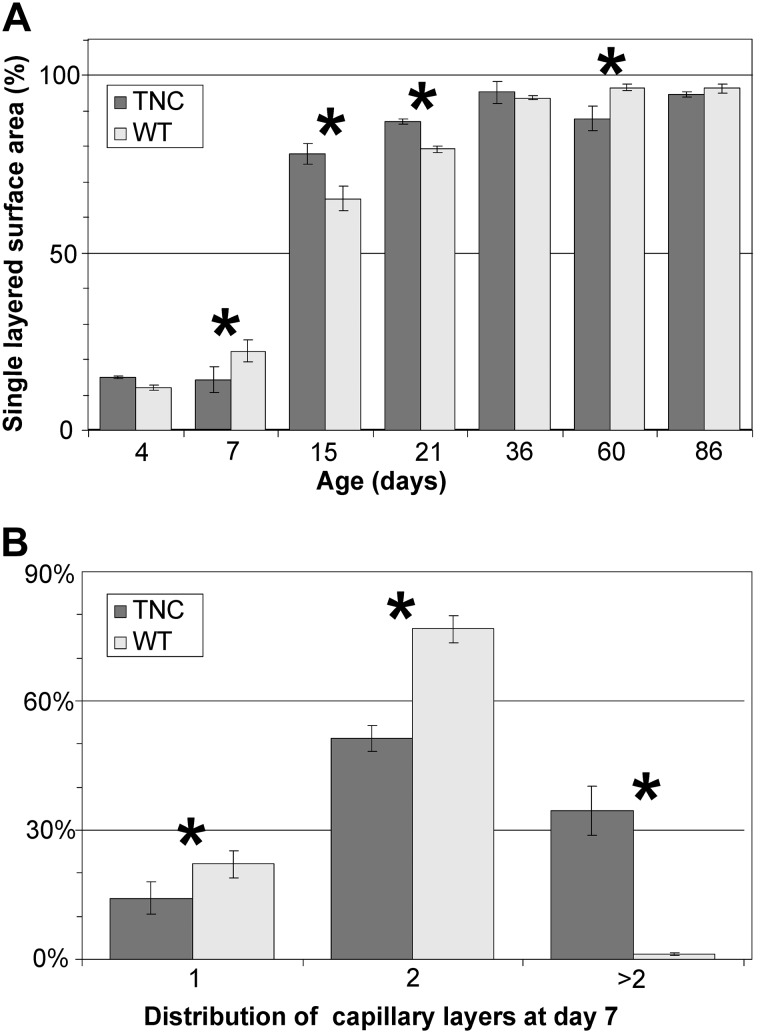
Microvascular maturation. In tenascin-C (TNC) null lungs, microvascular maturation was delayed and started after *day 7* (*A*). About one-third of the alveolar septa of TNC-deficient animals showed an atypical appearance with >2 capillary layers at *day 7* (*B*). At *days 15* and *21*, premature microvascular maturation was detected. The difference disappeared at *day 36*. At *day 60* a decreased fraction of single-layered septa was observed in TNC null lungs, but in adult animals at *day 86* no differences were detected (*A*). Error bars indicate SD; *n* = 5 lungs of male mice per time point and genotype. WT, wild type. *Statistically significant, α < 0.05.

**Fig. 6.**
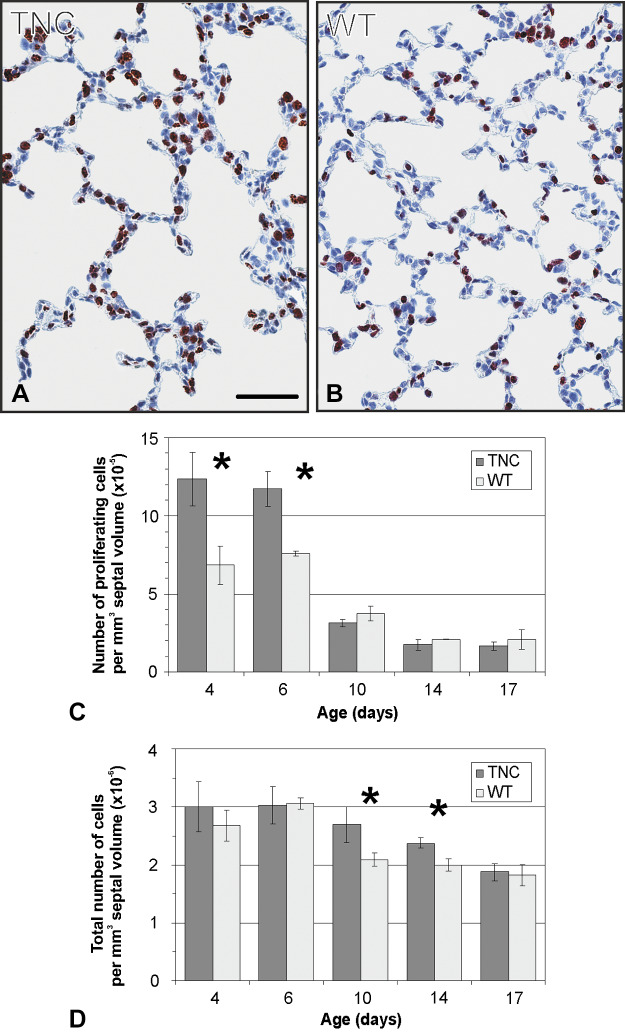
Cell proliferation and total number of cells. Lung sections of tenascin-C (TNC)-deficient and wild-type (WT) mice were stained with anti-Ki-67, a marker for cell proliferation, and counterstained with hematoxylin as shown for *postnatal day 6* (*A* and *B*). The number of Ki-67-positive cells (*C*) as well as the total number of cells (*D*) per mm^3^ of septal volume was evaluated between *postnatal days 4* and *17*. Both in wild-type and in TNC null lungs a peak of proliferating cells was detected at *days 4* and *6*, but the number of Ki-67-positive cells observed in mice lacking TNC exceeded that in wild-type (*C*). At *days 10* and *14* the total number of cells per mm^3^ was larger in TNC null than in wild-type lungs (*D*). Scale bar, 50 μm. Error bars indicate SD; *n* = 3 lungs of male mice per time point and genotype. *Statistically significant, α < 0.05.

By comparing the morphology of the inter-air space septa between wild-type and TNC null lungs using light and electron microscopy, focal areas of atypically thickened septa with an accumulation of capillaries and connective tissue, as well as an increased cellularity, were observed in TNC null lungs at *day 7* (Fig. 3). To better characterize this phenotype, *1*) the septal wall thickness was measured, *2*) microvascular maturation was followed by estimating the septal surface area possessing double- versus single-layered capillary networks on electron microscopical images using intersection counting, and *3*) the number of proliferating cells (Ki-67-positive cells) as well as *4*) the total number of cells was stereologically estimated. The mean septal wall thickness of TNC null lungs was increased by 100% at *day 7* (Fig. 4*A*). A histogram of the measured thickness revealed a shift from the classes of thinner measurements (0–10 μm) to thicker measurements (15–65 μm) in the TNC null lungs at this age (Fig. 4*B*). This result underlines our impression that only focal areas of the septa are thickened. Microvascular maturation was delayed and started after *day 7* in TNC null animals (Fig. 5). In addition, ~33% of the alveolar septa of the TNC null lungs showed an atypical appearance with >2 capillary layers at *day 7*. This phenotype was only observed in tenascin-C null lungs of this age. At *days 15* and *21*, microvascular maturation appeared to be overcompensated. The percentage of mature septa was increased in TNC null lungs, but the difference disappeared at *day 36*. At *day 60* a decreased fraction of mature septa was detected in TNC null mice. In adult animals at *day 86* no differences were observed (Fig. 5). Following cell proliferation by estimating the number of Ki-67-positive cells, a peak of proliferating cells was detected at *days 4* and *6* both in wild-type and TNC null animals. However, the number of proliferating cells was significantly larger in tenascin-C null lungs than in wild type (Fig. 6*C*). The total number of cells was increased in tenascin-C-deficient lungs at *days 10* and *14*, but at *day 17* or later no differences were observed (Fig. 6*D* and data not shown).

**Fig. 7.**
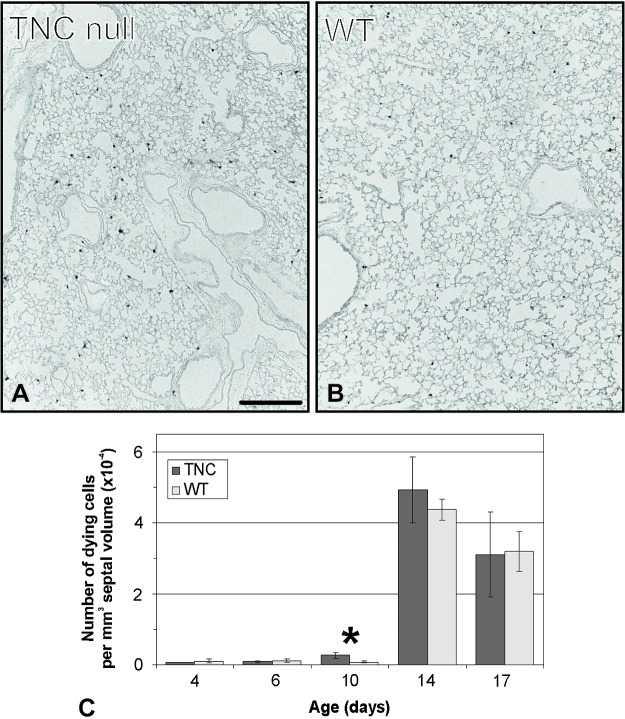
TUNEL assay-positive cells. Sections of tenascin-C (TNC) null and wild-type (WT) lungs were labeled by the TUNEL procedure as shown for *postnatal day 10* (*A* and *B*). The TUNEL assay stains cells possessing a large amount of DNA breakage, which is typical for programed cell death and highly elevated DNA repair. A peak of TUNEL-positive cells was observed at *days 14* and *17* in both TNC-deficient and wild-type lungs. At *day 10* a fourfold increase was detected in lungs lacking TNC compared with wild type (*C*). Scale bar, 200 μm. Error bars indicate SD; *n* = 3 lungs of male mice per time point and genotype. *Statistically significant, α < 0.05.

Asking whether this disappearance of the difference in the total normal number of cells at *day 17* or later may be explained by an increased rate of cell death, the number of TUNEL-positive cells was compared between TNC null and wild-type lungs. The TUNEL assay stains cells possessing a large amount of DNA breakage, which is typical for apoptosis, programed cell death, and highly elevated DNA repair. Both in wild-type and TNC null animals a peak of TUNEL-positive cells was observed at *days 14* and *17*. In addition, TNC-deficient lungs showed a premature increased rate of TUNEL-positive cells at *day 10*. At all other investigated time points no differences were observed (Fig. 7).

## DISCUSSION

**Fig. 8.**
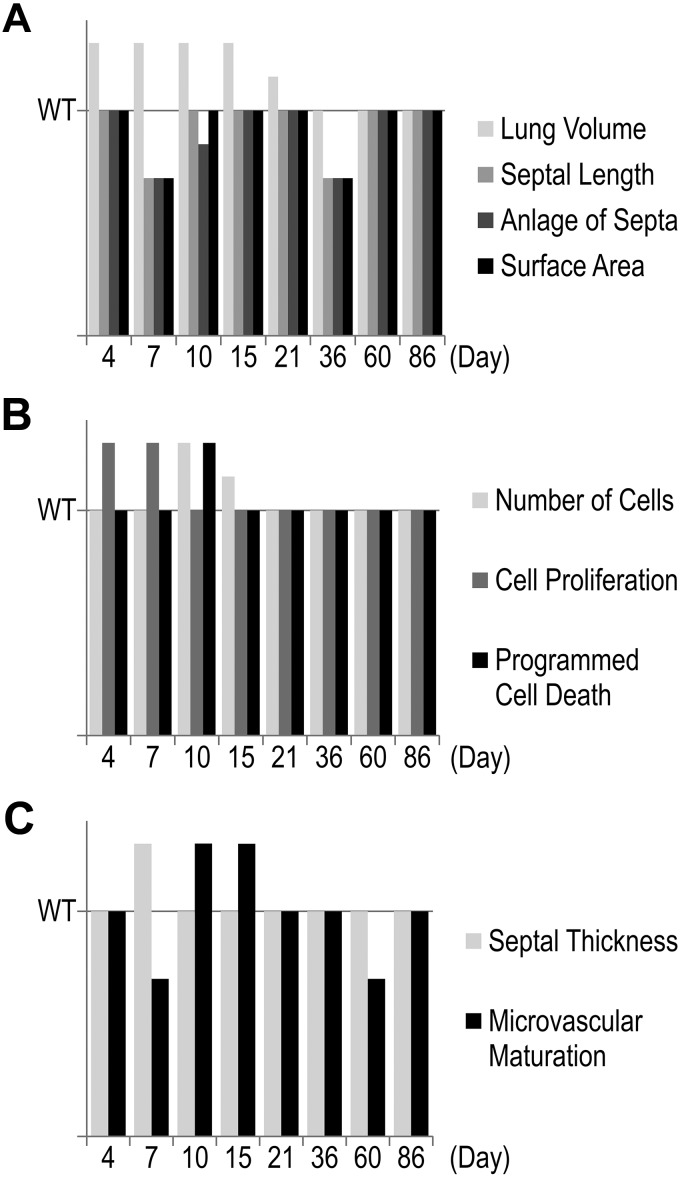
Timeline of phenotypes. The different phenotypes in tenascin-C (TNC)-deficient lungs are compared with the phenotype of wild-type (WT) lungs. *A* summarizes the structural differences: Lung volumes of TNC-deficient lungs are increased between *days 4* and *21*, but the length of the free septal edge, the anlage of septa, and the alveolar surface area are all decreased at *days 7* (to *day 10*) and *36*. The three of them catch up at *days 15–21* and a second time at *day 60*. *B* illustrates cell number, proliferation, and death. An increased cell proliferation at *days 4–7* is associated with an increased number of cells at *days 10–15* in TNC-deficient lungs. Programmed cell death is increased at *day 10*, which results in a normalization of the number of cells at *days 15–86* compared with wild type. *C* compares septal thickness and microvascular maturation. It seems to be that the delay in classical alveolarization at *day 7* causes an increase in the septal thickness. Microvascular maturation is also affected at *day 7* but showed a decreased maturity. The same was observed at *day 60*, which is most likely due to a catch-up alveolarization between *days 36* and *60*. All data are given as increase or decrease compared with wild type.

Although numerous reports on TNC expression during organ and tissue development exist and many in vitro studies have suggested multiple functions for this protein during development [see introduction, Jones and Jones (36), and Chiquet-Ehrismann et al. (9)], until now only one developmental abnormality has been reported in lungs of TNC-deficient mice (13, 60). Recently, we described a reduced branching morphogenesis during the development of the bronchial tree (60). In the present study we investigated the effect of TNC deficiency during postnatal lung development using the TNC null mouse strain of Forsberg et al. (24). Early postnatal lung development is characterized by the start of alveolarization and microvascular maturation as well as a peak of cell proliferation. It is well acknowledged that TNC expression peaks in the lung while these processes take place. By following the anlage of new septa and microvascular maturation by stereological estimations, we observed that both developmental processes were delayed in TNC null lungs and started after *day 7* (Figs. 2*B*, 5*A*, and 8), which is 3–4 days too late. This result lets us conclude that TNC contributes to the regulation of alveolarization/septation and microvascular maturation during early postnatal lung development. Remarkably, at *day 7* about one-third of the septal surface area present in TNC null lungs showed an atypical structure with >2 capillary layers (Fig. 5*B*). To the best of our knowledge, such incorrectly structured septa have never been detected during postnatal lung development before.

### TNC and Cell Migration

The expression of TNC is in part controlled by mechanical stimuli. Furthermore, the presence of TNC facilitates cell migration, e.g., by binding of TNC to the cell-binding domain of fibronectin or by the recognition of TNC by α8-integrin (8, 13, 15, 78). Since the process of lifting off of new alveolar septa most likely includes mechanical forces and requires a coordinated migration of all cell types present in the distal lung, this phenotype implies that cell migration and the transduction of mechanical forces may be impaired by TNC deficiency. In consequence, we hypothesize that TNC contributes to mechanotransduction and to the regulation of cell migration, which are both required for the lifting off of new alveolar septa and for the formation of correctly structured alveolar septa including the capillary network during early postnatal lung development.

### Cell Proliferation

In parallel with the delayed start of alveolarization and microvascular maturation, an increased number of proliferating cells was detected in TNC-deficient lungs at *days 4* and *6* (Fig. 6*C*). We therefore conclude that TNC also takes part in the regulation of cell proliferation and thus seems to be a key factor for the regulation of the major developmental processes, i.e., alveolarization, microvascular maturation, cell migration, and cell proliferation, taking place during early postnatal lung development. Moreover, our result of an increased cell proliferation is basically interesting, because this study is the first one detecting this phenomenon in vivo in TNC-deficient mice. Many in vitro studies have shown that depending on the cell type, TNC can either stimulate (11, 16, 35, 37, 72) or inhibit cell proliferation (11, 17, 55). However, in vivo studies have only observed a reduced rate of cell proliferation in TNC-deficient mice so far, namely, in association with a model of renal glomerulonephritis and in association with the behavior of neural precursor cells during the development of the central nervous system (28, 51). Thus, our results indicate that TNC can exert supportive or inhibitory effects on cell proliferation not only in vitro but also in vivo.

### Mechanical Forces

Taking into account that TNC expression may be induced because of mechanical strain and TNC is highly expressed at the tips of the alveolar septa, which are recognized to bear high mechanical forces, we moreover speculate that TNC expression might be upregulated by mechanical stimuli during early postnatal lung development. Since TNC seems to contribute to the regulation of cellular processes such as cell migration and cell proliferation during early postnatal lung development, its function during this period might be described as that of a mechanotransducer, in the sense that mechanical stimulation promotes cellular action via upregulation of TNC expression.

### Continued Alveolarization

The peak of TNC expression during the first postnatal week is followed by a decline to markedly reduced, but still detectable, levels during the third postnatal week during normal lung development in rats and mice (58, 63, 85). Unexpectedly, besides impairing early postnatal lung development (classical alveolarization), TNC deficiency did also alter later stages of lung development. In TNC-deficient lungs the continued alveolarization (2nd phase) was prolonged. While a premature microvascular maturation was detected at *days 15* and *21* (Figs. 2*B* and 5*A*), a delay of microvascular maturation was observed at *day 60*. The latter may be due to the catch-up of alveolarization observed between *days 36* and *60* in the TNC-deficient lungs, because newly formed septa are immature and it takes a short while until they mature. Therefore, we hypothesize that the observed higher “input rate” of new septa induces a higher percentage of immature septa in the TNC-deficient lungs at *day 60*. The altered continued alveolarization lets us hypothesize that not only does TNC contribute to the lifting off of new septa and microvascular maturation at the start of postnatal lung development but also the low levels of TNC detected during later stages of lung development contribute to the regulation of both of the latter-named processes.

### Programmed Cell Death

Given the roles of the protein TNC both during early postnatal lung development and during later stages that have been demonstrated in the present study, it seems to be surprising that no differences were detected in TNC-deficient lungs in adulthood at *postnatal day 86*. This result may be explained by the presence of corrective mechanisms during postnatal lung development in TNC-deficient animals. Although an increased number of proliferating cells was observed in TNC null lungs at *days 4* and *6*, the total number of cells was increased only at *days 10* and *14*, but not thereafter (Fig. 6). A possible mechanism explaining this phenomenon would be a compensatory alteration in the rate of programmed cell death similar to the alterations observed in rats that were treated with dexamethasone as neonates (45). Estimating the number of TUNEL-positive cells, a premature peak of positive cells was detected in TNC null animals at *day 10* (Fig. 7). Unfortunately, the TUNEL assay is not completely specific for programmed cell death. It also detects cells expressing a high amount of DNA breakage during DNA repair. Because programmed cell death is reported during this stage of development, it is likely that at least a high number of the TUNEL-positive cells are dying. The present study is the second one reporting that both cell proliferation and programmed cell death are altered in TNC-deficient mice during development. However, in contrast with our observations, Garcion et al. found a reduced rate of cell proliferation and a reduced rate of programmed cell death of neural precursor cells during development of the central nervous system in TNC-deficient mice (28). Thus, the compensation of impaired cell proliferation by the adaptation of programmed cell death seems to be an important corrective mechanism that leads to the apparent normality of TNC-deficient mice in adulthood. Furthermore, it needs to be mentioned that the present study is the first one following TUNEL-positive cells in the developing mouse lung. Over the last decades, postnatal lung development was generally considered to be identical in mice and rats. However, whereas rat lung development was well characterized by morphometrical methods, postnatal lung development in the mouse was only followed by morphological observations. We were recently able to show that postnatal lung development in mice and rats is not identical regarding the end point of alveolarization, the rate of the anlage of new alveolar septa, and the growth rate of the lung parenchyma (50, 70). In the present study we further observe that the peak of TUNEL-positive cells starts earlier in mice than in rats. Whereas in rats a peak was detected at *days 19* and *21* (45, 68), the peak was observed in mice already at *days 14* and *17* (Fig. 7).

### Rescue of Phenotype

An additional question arising is why the anlage of new alveolar septa and septal surface area observed in adult TNC-deficient animals is identical to wild type. The phenotype of a practically absent formation of new septa between *days 4* and *7* in TNC-deficient lungs was compensated in the TNC-deficient lungs leading to an increased rate of newly forming septa between *days 7* and *15*. The reduced formation of new septa between *days 21* and *36* in TNC-deficient animals was compensated by prolonged alveolarization between *days 36* and *60* (Fig. 2*B*). In parallel with the prolonged alveolarization a decrease in the fraction of the septal surface area characterized by a single-layered capillary network (mature capillary network) was observed between *days 36* and *60* in TNC-deficient lungs (Fig. 5*A*). Principally, the lifting off of new septa requires the existence of a double-layered capillary network. Whereas during classical alveolarization (1st phase), double-layered capillary networks are still present in the prenatally formed septa, the late formation of new septa is facilitated by local duplications of the single-layered capillary network at the sides of septation (65, 70). Most likely, the double-layered capillary networks that are present during adolescence and in young adults in wild-type and TNC-deficient lungs (Fig. 5*A*) appear at sites where new septa are forming and grow into the alveolar lumen. Therefore, it is anticipated that an increased rate of formation of new septa should result in a transient immaturity of the alveolar septa.

Another process that was associated with TNC deficiency during postnatal lung development and that practically represents a rescue mechanism is the increased lung volume we observed in TNC null animals during early postnatal lung development (Fig. 2*A*). Although in TNC null lungs practically no septa were formed between *days 4* and *7* and the anlage of septa was still decreased at *day 10*, the septal surface area of TNC-deficient animals was only slightly reduced at *day 7* and not significantly different at *day 10* (Fig. 2*C*). However, this compensatory effect was restricted to the first 3 postnatal weeks and did not rescue the reduced formation of new alveolar septa between *days 21* and *36*.

In principle, the increase in the lung volume could be caused by two different effects: increased lung growth and/or increased compliance of the lung tissue and the thorax (the filling of the lungs was done when the thorax was still closed except a small hole in the diaphragm). We would not like to speculate as to which of the mechanisms is predominately involved. However, for the TNC-deficient mice it does not matter, because in both cases, lung growth versus compliance, a larger gas exchange area could be used compared with wild-type—and most likely the latter is what counts for the mice.

Does the question of increased lung growth versus lung compliance affect the stereological estimations? No, because the estimations are done as well as possible under standardized conditions. Using a constant pressure for filling represents the state of the art (34). However, stereological estimations do not tell us anything about the reason why a parameter is different in different groups of animals.

Does the question affect the finding of delayed and catch-up alveolarization? No, because without the observed increase in lung volume the effect would be even more pronounced.

### Conclusion

In summary, we describe a new developmental phenotype of the TNC null mouse. In TNC-deficient lungs both alveolarization and microvascular maturation started with a delay, cell proliferation was increased, and thick septa with an accumulation of capillaries and cells were observed during early postnatal lung development. These results are summarized in Fig. 8. These results let us hypothesize that TNC contributes to the lifting off of new septa, to the regulation of cell migration and cell proliferation, and furthermore to microvascular maturation at the start of postnatal lung development. The increased cell proliferation was most likely rescued by an increased number of dying cells (TUNEL-positive cells), whereas the delayed alveolarization and microvascular maturation were compensated by an increase in the formation of new alveolar septa and an increase in septal maturation. In addition, the phase of continued alveolarization (2nd phase) was prolonged and, in parallel, the alveolar microvascular was less mature in TNC-deficient mice toward the end of continued alveolarization. The latter may be explained by an increased or better catch-up formation of new alveolar septa, which are immature directly after they are formed. We hypothesize that TNC contributes to the lifting off of new alveolar septa and microvascular maturation not only during early postnatal lung development but also during later stages.

## GRANTS

We are thankful for the support from Swiss National Science Foundation Grants 3100.068256.02, 3100A0-109874, 310030-153468, and 310030_175953.

## DISCLOSURES

No conflicts of interest, financial or otherwise, are declared by the authors.

## AUTHOR CONTRIBUTIONS

J.C.S. conceived and designed research; S.I.M. and J.C.S. performed experiments; S.I.M. and J.C.S. analyzed data; S.I.M. and J.C.S. interpreted results of experiments; S.I.M. and J.C.S. prepared figures; S.I.M. drafted manuscript; S.I.M. and J.C.S. edited and revised manuscript; S.I.M. and J.C.S. approved final version of manuscript.

## References

[1] Ackermann M, Houdek JP, Gibney BC, Ysasi A, Wagner W, Belle J, Schittny JC, Enzmann F, Tsuda A, Mentzer SJ, Konerding MA Sprouting and intussusceptive angiogenesis in postpneumonectomy lung growth: mechanisms of alveolar neovascularization. Angiogenesis 17: 541–551, 2014. doi:10.1007/s10456-013-9399-9. 24150281 PMC4061467

[2] Altman PA, Dittmer DS Biology Data Book. Bethesda, MD: Federation of American Societies for Experimental Biology, 1972.

[3] Amy RW, Bowes D, Burri PH, Haines J, Thurlbeck WM Postnatal growth of the mouse lung. J Anat 124: 131–151, 1977. 914698 PMC1235518

[4] Barré SF, Haberthür D, Cremona TP, Stampanoni M, Schittny JC The total number of acini remains constant throughout postnatal rat lung development. Am J Physiol Lung Cell Mol Physiol 311: L1082–L1089, 2016. doi:10.1152/ajplung.00325.2016. 27760763

[5] Bartsch U The extracellular matrix molecule tenascin-C: expression in vivo and functional characterization in vitro. Prog Neurobiol 49: 145–161, 1996. doi:10.1016/0301-0082(96)00014-7. 8844824

[6] Burri PH The postnatal growth of the rat lung. 3. Morphology. Anat Rec 180: 77–98, 1974. doi:10.1002/ar.1091800109. 4416419

[7] Burri PH, Dbaly J, Weibel ER The postnatal growth of the rat lung. I. Morphometry. Anat Rec 178: 711–730, 1974. doi:10.1002/ar.1091780405. 4592625

[8] Chiquet-Ehrismann R, Chiquet M Tenascins: regulation and putative functions during pathological stress. J Pathol 200: 488–499, 2003. doi:10.1002/path.1415. 12845616

[9] Chiquet-Ehrismann R, Hagios C, Schenk S The complexity in regulating the expression of tenascins. BioEssays 17: 873–878, 1995. doi:10.1002/bies.950171009. 7487968

[10] Chiquet-Ehrismann R, Kalla P, Pearson CA, Beck K, Chiquet M Tenascin interferes with fibronectin action. Cell 53: 383–390, 1988. doi:10.1016/0092-8674(88)90158-4. 2452695

[11] Chiquet-Ehrismann R, Mackie EJ, Pearson CA, Sakakura T Tenascin: an extracellular matrix protein involved in tissue interactions during fetal development and oncogenesis. Cell 47: 131–139, 1986. doi:10.1016/0092-8674(86)90374-0. 2428505

[12] Chiquet-Ehrismann R, Orend G, Chiquet M, Tucker RP, Midwood KS Tenascins in stem cell niches. Matrix Biol 37: 112–123, 2014. doi:10.1016/j.matbio.2014.01.007. 24472737

[13] Chiquet-Ehrismann R, Tucker RP Tenascins and the importance of adhesion modulation. Cold Spring Harb Perspect Biol 3: a004960, 2011. doi:10.1101/cshperspect.a004960. 21441591 PMC3101840

[15] Chiquet M, Matthisson M, Koch M, Tannheimer M, Chiquet-Ehrismann R Regulation of extracellular matrix synthesis by mechanical stress. Biochem Cell Biol 74: 737–744, 1996. doi:10.1139/o96-080. 9164643

[16] Chung CY, Murphy-Ullrich JE, Erickson HP Mitogenesis, cell migration, and loss of focal adhesions induced by tenascin-C interacting with its cell surface receptor, annexin II. Mol Biol Cell 7: 883–892, 1996. doi:10.1091/mbc.7.6.883. 8816995 PMC275940

[17] Crossin KL Cytotactin binding: inhibition of stimulated proliferation and intracellular alkalinization in fibroblasts. Proc Natl Acad Sci USA 88: 11403–11407, 1991. doi:10.1073/pnas.88.24.11403. 1722330 PMC53143

[18] Crossin KL Tenascin: a multifunctional extracellular matrix protein with a restricted distribution in development and disease. J Cell Biochem 61: 592–598, 1996. doi:10.1002/(SICI)1097-4644(19960616)61:4<592:AID-JCB13>3.0.CO;2-I. 8806083

[19] Cruz-Orive LM, Weibel ER Sampling designs for stereology. J Microsc 122: 235–257, 1981. doi:10.1111/j.1365-2818.1981.tb01265.x. 7017151

[20] Dubreuil G, Lacoste A, Raymond R Observations sur le développement du poumon humain. Bull Histol Tech Microsc 13: 235–245, 1936.

[21] Ekblom P, Aufderheide E Stimulation of tenascin expression in mesenchyme by epithelial-mesenchymal interactions. Int J Dev Biol 33: 71–79, 1989. 2484680

[22] Erhardt W, Henke J, Haberstroh J, Baumgartner C, Tacke S Analgesie. In: Anästhesie und Analgesie beim Klein- und Heimtier. Stuttgart, Germany: Schattauer, 2011, p. 383–434.

[23] Erickson HP, Bourdon MA Tenascin: an extracellular matrix protein prominent in specialized embryonic tissues and tumors. Annu Rev Cell Biol 5: 71–92, 1989. doi:10.1146/annurev.cb.05.110189.000443. 2480799

[24] Forsberg E, Hirsch E, Fröhlich L, Meyer M, Ekblom P, Aszodi A, Werner S, Fässler R Skin wounds and severed nerves heal normally in mice lacking tenascin-C. Proc Natl Acad Sci USA 93: 6594–6599, 1996. doi:10.1073/pnas.93.13.6594. 8692862 PMC39070

[25] Frasca JM, Parks VR A routine technique for double-staining ultrathin sections using uranyl and lead salts. J Cell Biol 25: 157–161, 1965. doi:10.1083/jcb.25.1.157. 14289358 PMC2106611

[26] Fukamauchi F, Mataga N, Wang YJ, Sato S, Youshiki A, Kusakabe M Abnormal behavior and neurotransmissions of tenascin gene knockout mouse. Biochem Biophys Res Commun 221: 151–156, 1996. doi:10.1006/bbrc.1996.0561. 8660327

[27] Galilei G. Discorsi e Dimostrazioni Matematiche, Intorno à Due Nuove Scienze Attenenti alla Mecanica & i Movimenti Locali. Leiden, The Netherlands: Elsevier, 1638.

[28] Garcion E, Faissner A, ffrench-Constant C Knockout mice reveal a contribution of the extracellular matrix molecule tenascin-C to neural precursor proliferation and migration. Development 128: 2485–2496, 2001. 11493565 10.1242/dev.128.13.2485

[29] Gavrieli Y, Sherman Y, Ben-Sasson SA Identification of programmed cell death in situ via specific labeling of nuclear DNA fragmentation. J Cell Biol 119: 493–501, 1992. doi:10.1083/jcb.119.3.493. 1400587 PMC2289665

[30] Haberthür D, Barré SF, Tschanz SA, Yao E, Stampanoni M, Schittny JC Visualization and stereological characterization of individual rat lung acini by high-resolution X-ray tomographic microscopy. J Appl Physiol (1985) 115: 1379–1387, 2013. doi:10.1152/japplphysiol.00642.2013. 23970533

[31] Haberthür D, Hintermüller C, Marone F, Schittny JC, Stampanoni M Radiation dose optimized lateral expansion of the field of view in synchrotron radiation X-ray tomographic microscopy. J Synchrotron Radiat 17: 590–599, 2010. doi:10.1107/S0909049510019618. 20724780 PMC2927902

[32] Hendaoui I, Tucker RP, Zingg D, Bichet S, Schittny J, Chiquet-Ehrismann R Tenascin-C is required for normal Wnt/β-catenin signaling in the whisker follicle stem cell niche. Matrix Biol 40: 46–53, 2014. doi:10.1016/j.matbio.2014.08.017. 25196097

[33] Howard CV, Reed MG Unbiased Stereology. Three-Dimensional Measurement in Microscopy. Abingdon, UK: Garland Science/BIOS Scientific, 2005.

[34] Hsia CC, Hyde DM, Ochs M, Weibel ER; ATS/ERS Joint Task Force on Quantitative Assessment of Lung Structure An official research policy statement of the American Thoracic Society/European Respiratory Society: standards for quantitative assessment of lung structure. Am J Respir Crit Care Med 181: 394–418, 2010. doi:10.1164/rccm.200809-1522ST. 20130146 PMC5455840

[35] Huang W, Chiquet-Ehrismann R, Moyano JV, Garcia-Pardo A, Orend G Interference of tenascin-C with syndecan-4 binding to fibronectin blocks cell adhesion and stimulates tumor cell proliferation. Cancer Res 61: 8586–8594, 2001. 11731446

[36] Jones FS, Jones PL The tenascin family of ECM glycoproteins: structure, function, and regulation during embryonic development and tissue remodeling. Dev Dyn 218: 235–259, 2000. doi:10.1002/(SICI)1097-0177(200006)218:2<235:AID-DVDY2>3.0.CO;2-G. 10842355

[37] Jones PL, Crack J, Rabinovitch M Regulation of tenascin-C, a vascular smooth muscle cell survival factor that interacts with the α_v_β_3_ integrin to promote epidermal growth factor receptor phosphorylation and growth. J Cell Biol 139: 279–293, 1997. doi:10.1083/jcb.139.1.279. 9314546 PMC2139818

[38] Kaarteenaho-Wiik R, Kinnula V, Herva R, Pääkkö P, Pöllänen R, Soini Y Distribution and mRNA expression of tenascin-C in developing human lung. Am J Respir Cell Mol Biol 25: 341–346, 2001. doi:10.1165/ajrcmb.25.3.4460. 11588012

[39] Kauffman SL, Burri PH, Weibel ER The postnatal growth of the rat lung. II. Autoradiography. Anat Rec 180: 63–76, 1974. doi:10.1002/ar.1091800108. 4416418

[40] Kiernan BW, Garcion E, Ferguson J, Frost EE, Torres EM, Dunnett SB, Saga Y, Aizawa S, Faissner A, Kaur R, Franklin RJ, ffrench-Constant C Myelination and behaviour of tenascin-C null transgenic mice. Eur J Neurosci 11: 3082–3092, 1999. doi:10.1046/j.1460-9568.1999.00729.x. 10510172

[41] Koch M, Wehrle-Haller B, Baumgartner S, Spring J, Brubacher D, Chiquet M Epithelial synthesis of tenascin at tips of growing bronchi and graded accumulation in basement membrane and mesenchyme. Exp Cell Res 194: 297–300, 1991. doi:10.1016/0014-4827(91)90368-5. 1709104

[42] Lovric G, Barré SF, Schittny JC, Roth-Kleiner M, Stampanoni M, Mokso R Dose optimization approach to fast X-ray microtomography of the lung alveoli. J Appl Cryst 46: 856–860, 2013. doi:10.1107/S0021889813005591. 24046488 PMC3769076

[43] Lovric G, Mokso R, Arcadu F, Vogiatzis Oikonomidis I, Schittny JC, Roth-Kleiner M, Stampanoni M Tomographic in vivo microscopy for the study of lung physiology at the alveolar level. Sci Rep 7: 12545, 2017. doi:10.1038/s41598-017-12886-3. 28970505 PMC5624921

[44] Lovric G, Vogiatzis Oikonomidis I, Mokso R, Stampanoni M, Roth-Kleiner M, Schittny JC Automated computer-assisted quantitative analysis of intact murine lungs at the alveolar scale. PLoS One 12: e0183979, 2017. doi:10.1371/journal.pone.0183979. 28934236 PMC5608210

[45] Luyet C, Burri PH, Schittny JC Suppression of cell proliferation and programmed cell death by dexamethasone during postnatal lung development. Am J Physiol Lung Cell Mol Physiol 282: L477–L483, 2002. doi:10.1152/ajplung.00406.2000. 11839541

[46] Mackie EJ Molecules in focus: tenascin-C. Int J Biochem Cell Biol 29: 1133–1137, 1997. doi:10.1016/S1357-2725(97)00031-9. 9438376

[47] Mackie EJ, Tucker RP The tenascin-C knockout revisited. J Cell Sci 112: 3847–3853, 1999. 10547346 10.1242/jcs.112.22.3847

[48] Matsuda A, Yoshiki A, Tagawa Y, Matsuda H, Kusakabe M Corneal wound healing in tenascin knockout mouse. Invest Ophthalmol Vis Sci 40: 1071–1080, 1999. 10235540

[49] Michel RP, Cruz-Orive LM Application of the Cavalieri principle and vertical sections method to lung: estimation of volume and pleural surface area. J Microsc 150: 117–136, 1988. doi:10.1111/j.1365-2818.1988.tb04603.x. 3411604

[50] Mund SI, Stampanoni M, Schittny JC Developmental alveolarization of the mouse lung. Dev Dyn 237: 2108–2116, 2008. doi:10.1002/dvdy.21633. 18651668

[51] Nakao N, Hiraiwa N, Yoshiki A, Ike F, Kusakabe M Tenascin-C promotes healing of Habu-snake venom-induced glomerulonephritis: studies in knockout congenic mice and in culture. Am J Pathol 152: 1237–1245, 1998. 9588892 PMC1858571

[52] Ohta M, Sakai T, Saga Y, Aizawa S, Saito M Suppression of hematopoietic activity in tenascin-C-deficient mice. Blood 91: 4074–4083, 1998. 9596652

[53] Olave N, Lal CV, Halloran B, Pandit K, Cuna AC, Faye-Petersen OM, Kelly DR, Nicola T, Benos PV, Kaminski N, Ambalavanan N Regulation of alveolar septation by microRNA-489. Am J Physiol Lung Cell Mol Physiol 310: L476–L487, 2016. doi:10.1152/ajplung.00145.2015. 26719145 PMC4773841

[54] Orend G, Chiquet-Ehrismann R Adhesion modulation by antiadhesive molecules of the extracellular matrix. Exp Cell Res 261: 104–110, 2000. doi:10.1006/excr.2000.5041. 11082280

[55] Orend G, Huang W, Olayioye MA, Hynes NE, Chiquet-Ehrismann R Tenascin-C blocks cell-cycle progression of anchorage-dependent fibroblasts on fibronectin through inhibition of syndecan-4. Oncogene 22: 3917–3926, 2003. doi:10.1038/sj.onc.1206618. 12813465

[56] Reynolds ES The use of lead citrate at high pH as an electron-opaque stain in electron microscopy. J Cell Biol 17: 208–212, 1963. doi:10.1083/jcb.17.1.208. 13986422 PMC2106263

[57] Riffenburgh RH Statistics in Medicine. San Diego: Academic, 1999.

[58] Roth-Kleiner M, Berger TM, Gremlich S, Tschanz SA, Mund SI, Post M, Stampanoni M, Schittny JC Neonatal steroids induce a down-regulation of tenascin-C and elastin and cause a deceleration of the first phase and an acceleration of the second phase of lung alveolarization. Histochem Cell Biol 141: 75–84, 2014. doi:10.1007/s00418-013-1132-7. 23912843

[59] Roth-Kleiner M, Berger TM, Tarek MR, Burri PH, Schittny JC Neonatal dexamethasone induces premature microvascular maturation of the alveolar capillary network. Dev Dyn 233: 1261–1271, 2005. doi:10.1002/dvdy.20447. 15937935

[60] Roth-Kleiner M, Hirsch E, Schittny JC Fetal lungs of tenascin-C-deficient mice grow well, but branch poorly in organ culture. Am J Respir Cell Mol Biol 30: 360–366, 2004. doi:10.1165/rcmb.2002-0266OC. 12904321

[61] Roth-Kleiner M, Post M Similarities and dissimilarities of branching and septation during lung development. Pediatr Pulmonol 40: 113–134, 2005. doi:10.1002/ppul.20252. 15965895

[62] Saga Y, Yagi T, Ikawa Y, Sakakura T, Aizawa S Mice develop normally without tenascin. Genes Dev 6: 1821–1831, 1992. doi:10.1101/gad.6.10.1821. 1383086

[63] Sage EH, Bornstein P Extracellular proteins that modulate cell-matrix interactions. SPARC, tenascin, and thrombospondin. J Biol Chem 266: 14831–14834, 1991. 1714444

[64] Scherle W A simple method for volumetry of organs in quantitative stereology. Mikroskopie 26: 57–60, 1970. 5530651

[65] Schittny JC Development of the lung. Cell Tissue Res 367: 427–444, 2017. doi:10.1007/s00441-016-2545-0. 28144783 PMC5320013

[66] Schittny JC How high resolution 3-dimensional imaging changes our understanding of postnatal lung development. Histochem Cell Biol 150: 677–691, 2018. doi:10.1007/s00418-018-1749-7. 30390117 PMC6267404

[67] Schittny JC, Burri PH Morphogenesis of the mammalian lung: aspects of structure and extracellular matrix components. In: Lung Development and Regeneration, edited by Massaro D, Massaro GD, Chambon P New York: Marcel Dekker, 2004, p. 275–317.

[68] Schittny JC, Djonov V, Fine A, Burri PH Programmed cell death contributes to postnatal lung development. Am J Respir Cell Mol Biol 18: 786–793, 1998. doi:10.1165/ajrcmb.18.6.3031. 9618383

[70] Schittny JC, Mund SI, Stampanoni M Evidence and structural mechanism for late lung alveolarization. Am J Physiol Lung Cell Mol Physiol 294: L246–L254, 2008. doi:10.1152/ajplung.00296.2007. 18032698

[71] Scholzen T, Gerdes J The Ki-67 protein: from the known and the unknown. J Cell Physiol 182: 311–322, 2000. doi:10.1002/(SICI)1097-4652(200003)182:3<311:AID-JCP1>3.0.CO;2-9. 10653597

[72] Seiffert M, Beck SC, Schermutzki F, Müller CA, Erickson HP, Klein G Mitogenic and adhesive effects of tenascin-C on human hematopoietic cells are mediated by various functional domains. Matrix Biol 17: 47–63, 1998. doi:10.1016/S0945-053X(98)90124-X. 9628252

[73] Shinohara Y, Okamoto K, Goh Y, Kiga N, Tojyo I, Fujita S Inhibition of fibrous adhesion formation in the temporomandibular joint of tenascin-C knockout mice. Eur J Histochem 58: 2337, 2014. doi:10.4081/ejh.2014.2337. 25578971 PMC4289843

[74] Stampanoni M, Borchert G, Wyss P, Abela R, Patterson B, Hunt S, Vermeulen D, Ruegsegger P High resolution X-ray detector for synchrotron-based microtomography. Nucl Instrum Meth A 491: 291–301, 2002. doi:10.1016/S0168-9002(02)01167-1.

[75] Stampanoni M, Groso A, Isenegger A, Mikuljan Q, Chen Q, Bertrand A, Henein S, Betemps R, Frommherz U, Böhler P, Meister D, Lange M, Abela R Trends in synchrotron-based tomographic imaging: the SLS experience. In: Proceedings of SPIE, Developments in X-Ray Tomography V, edited by Bonse U Bellingham, WA: SPIE, 2006, vol. 6318, p. 1605–7422.

[76] Sterio DC The unbiased estimation of number and sizes of arbitrary particles using the disector. J Microsc 134: 127–136, 1984. doi:10.1111/j.1365-2818.1984.tb02501.x. 6737468

[77] Tschanz SA, Salm LA, Roth-Kleiner M, Barré SF, Burri PH, Schittny JC Rat lungs show a biphasic formation of new alveoli during postnatal development. J Appl Physiol (1985) 117: 89–95, 2014. doi:10.1152/japplphysiol.01355.2013. 24764134

[78] Tucker RP, Chiquet-Ehrismann R Tenascin-C: its functions as an integrin ligand. Int J Biochem Cell Biol 65: 165–168, 2015. doi:10.1016/j.biocel.2015.06.003. 26055518

[79] Tucker RP, Ferralli J, Schittny JC, Chiquet-Ehrismann R Tenascin-C and tenascin-W in whisker follicle stem cell niches: possible roles in regulating stem cell proliferation and migration. J Cell Sci 126: 5111–5115, 2013. doi:10.1242/jcs.134650. 24101721

[80] Vollmer G Biologic and oncologic implications of tenascin-C/hexabrachion proteins. Crit Rev Oncol Hematol 25: 187–210, 1997. doi:10.1016/S1040-8428(97)00004-8. 9177941

[81] Vuckovic A, Herber-Jonat S, Flemmer AW, Roubliova XI, Jani JC Alveolarization genes modulated by fetal tracheal occlusion in the rabbit model for congenital diaphragmatic hernia: a randomized study. PLoS One 8: e69210, 2013. doi:10.1371/journal.pone.0069210. 23840910 PMC3698086

[82] Weibel ER Morphometric and stereological methods in respiratory physiology including fixation techniques. In: Techniques in Respiratory Physiology: Part I. Shannon, Ireland: Elsevier Scientific, 1984, p. 1–35.

[83] Woods JC, Schittny JC Lung structure at preterm and term birth. In: Fetal Lung Development: Clinical Correlates & Future Technologies, edited by Jobe AH, Whitsett JA, Abman SH New York: Cambridge University Press, 2016, p. 126–140.

[84] Yoshida T, Akatsuka T, Imanaka-Yoshida K Tenascin-C and integrins in cancer. Cell Adhes Migr 9: 96–104, 2015. doi:10.1080/19336918.2015.1008332. 25793576 PMC4422796

[85] Young SL, Chang LY, Erickson HP Tenascin-C in rat lung: distribution, ontogeny and role in branching morphogenesis. Dev Biol 161: 615–625, 1994. doi:10.1006/dbio.1994.1057. 7508872

[86] Zeltner TB, Bertacchini M, Messerli A, Burri PH Morphometric estimation of regional differences in the rat lung. Exp Lung Res 16: 145–158, 1990. doi:10.3109/01902149009087879. 2328712

[87] Zeltner TB, Burri PH The postnatal development and growth of the human lung. II. Morphology. Respir Physiol 67: 269–282, 1987. doi:10.1016/0034-5687(87)90058-2. 3575906

[88] Zhao Y, Young SL Tenascin in rat lung development: in situ localization and cellular sources. Am J Physiol Lung Cell Mol Physiol 269: L482–L491, 1995. doi:10.1152/ajplung.1995.269.4.L482. 7485520

